# What makes gouty inflammation so variable?

**DOI:** 10.1186/s12916-017-0922-5

**Published:** 2017-08-18

**Authors:** Robert Terkeltaub

**Affiliations:** 1 VA San Diego Healthcare System, 111K, 3350 La Jolla Village Drive, San Diego, CA 92161 USA; 2 Department of Medicine, University of California San Diego, San Diego, CA USA

**Keywords:** Gout flare, NLRP3 inflammasome, AMPK, PPAR-γ, PGC1B, Short-chain fatty acids, β-hydroxybutyrate, miR-146a, miR-155, IL-37

## Abstract

Acute gout arthritis flares contribute dominantly to gout-specific impaired health-related quality of life, representing a progressively increasing public health problem. Flares can be complex and expensive to treat, partly due to the frequent comorbidities. Unmet needs in gout management are more pressing given the markedly increasing gout flare hospital admission rates. In addition, chronic gouty arthritis can cause joint damage and functional impairment. This review addresses new knowledge on the basis for the marked, inherent variability of responses to deposited urate crystals, including the unpredictable and self-limited aspects of many gout flares. Specific topics reviewed include how innate immunity and two-signal inflammasome activation intersect with diet, metabolism, nutritional biosensing, the microbiome, and the phagocyte cytoskeleton and cell fate. The paper discusses the roles of endogenous constitutive regulators of inflammation, including certain nutritional biosensors, and emerging genetic and epigenetic factors. Recent advances in the basis of variability in responses to urate crystals in gout provide information about inflammatory arthritis, and have identified potential new targets and strategies for anti-inflammatory prevention and treatment of gouty arthritis.

## Background

Acute gouty arthritis is a major and increasing public health problem given the substantial rise in gout prevalence over the last two decades [1]. Acute gout flares are the major contributor to gout-specific impaired health-related quality of life [2]. In this context, flares can be complex and expensive to treat [3], partly due to the commonly associated comorbidities, including cardiovascular disease, chronic kidney disease, and diabetes [4], and to frequent contraindications to one or more of the standard oral anti-inflammatory therapy options for gout (i.e., NSAIDs, colchicine, corticosteroids) [5]. Moreover, responses to these oral agents often take at least 12–24 hours to be robust, and can take from several days to a week to be complete [6]. Importantly, there has been a marked, progressive increase in hospitalizations due to gout flares over the last two decades, in stark contrast to the data for decreased hospitalizations specifically for poorly controlled flares of rheumatoid arthritis [7]. The capacity of chronic gouty arthritis to cause joint damage and functional impairment further adds to the societal and financial impact of the disease.

The pathogenesis of gouty inflammation is relatively well understood [8–10]. It is known that urate crystals promote cleavage of C5 on their surface, catalyzing the generation of C5a and C5b-9 involved in gouty inflammation [8]. There is also extensive knowledge of how the crystals activate phagocytes, the role of inflammation signaling and transcription factors, including mitogen activated protein kinases and AP-1, the role of the master transcriptional regulator NF-κB [8], and on the activation of the NACHT-LRR-PYD-containing protein 3 (NLRP3) inflammasome, with consequent IL-1β maturation and secretion [10, 11]. Many other urate crystal-induced inflammatory mediators are well characterized, including multiple prostaglandins, several leukotrienes (e.g., leukotriene B4) [8, 12], and a host of cytokines, including IL-8 and many other chemokines, IL-6, and TNF-α [8, 13]. Other noteworthy background observations include the late dampening effects of TGF-β in experimental acute gout [8]. This review focuses on the most recent research, which sheds new light on why human host responses to tissue deposits of urate crystals in gout are so variable in intensity, change in frequency between seasons, are so often unpredictable in onset, and are characteristically self-limited. The rapidly emerging research in this area points to multiple new targets and strategies for the unmet needs in the optimal prevention and treatment of gouty arthritis, as well as providing valuable lessons about innate immunity in rheumatic disease in general.

## Urate crystal deposits often remain clinically silent

Advanced imaging approaches, using high resolution ultrasound and highly specific dual energy CT (DECT), have emerged as extremely sensitive and specific diagnostic tools for urate crystal deposition in gout. A striking observation in several studies with each of these imaging modalities is that many of those with asymptomatic hyperuricemia, and not simply with very high levels of serum urate, have evidence for urate crystal deposition in articular as well as in certain periarticular tissue loci [14, 15]. For example, in a recent study [14], first metatarsophalangeal joint ultrasound was positive for urate crystal deposition in 36% of patients with asymptomatic hyperuricemia (defined as serum urate ≥ 6.9 mg/dL), but without substantial evidence of synovitis and bone erosion. In another study of asymptomatic hyperuricemia [15], DECT revealed urate crystal deposits in 24% of patients. DECT analyses demonstrate that tendon urate crystal deposits, which resolve relatively slowly with aggressive urate-lowering therapy (ULT) [16], are a marker for symptomatic gout [15]. However, ‘early gout’ (defined as ≤ 3 years disease duration) and well-established ‘late gout’, both with mean serum 7–7.5 mg/dL, have similar crystal volumes in joints [15].

Novel findings, using DECT, have further indicated that, in the absence of gout, urate crystals deposit in costal cartilages and intervertebral disks in middle-aged men [17], and at several sites of axial and peripheral joint involvement in spondyloarthropathy and seronegative rheumatoid arthritis patients [18, 19]. Nevertheless, the mechanism and tissue effects of deposited urate crystals in these conditions are unknown. Another provocative study suggested the formation of urate crystals, studied ex vivo, in circulating phagocytes of markedly hyperuricemic human blood [20].

Volumetric analysis of articular urate crystal deposits by DECT has suggested that there may be ‘threshold effects’ for expression of the gouty arthritis phenotype, with greater urate crystal deposit volume detectable in gouty joints compared to asymptomatic hyperuricemia [15]. However, in patients with gout, both exogenous and host factors are arrayed in opposition to promote and limit inflammatory responses to significant volumes of deposited urate crystals, including at specific sites in the joint (summarized in Table 1, and discussed below). The balance between these factors likely plays a core role in determining the expression of the gouty arthritis clinical phenotype.

**Table 1:** Factors regulating gouty inflammation as elucidated by recent research

Factors promoting acute inflammation in response to urate crystals		Factors limiting initiation of acute crustal-induced inflammation (with or without promoting resolution)	
Endogenous	Exogenous	Endogenous	Exogenous
1^st^ signal NLRP3 inflammasome activation: - C5a - GM-CSF - S100A8/A9	1^st^ signal NLRP3 inflammasome activation: - Long chain saturated fatty acids (e.g., palmitate) - Spikes in systemic acetate levels (e.g., via alcohol) Angiotensin converting enzyme inhibition: (via kinin B1 receptors)	Nutritional biosensing: - AMPK activity - PPAR-γ activity Microbiome (via production of short chain fatty acids)	Inhibitors of NLRP3 inflammasome activation: - Omega-3 fatty acids - Ketogenic diet (via β-hydroxybutyrate) Dietary fiber and short chain fatty acids (e.g., acetate, butyrate)
Genetic: - Common *PPARGC1B* risk allele A rs45520937 - Certain *CARD8*, *CD14*, *ILB* SNPs Epigenetic: - Certain Class I HDACs -miR-155	–	Possible Genetic Factors Epigenetic: - miR-146a	–
Other: - Changes in urate crystal morphology and surface constituents - Hyperuricemia (via decreased phagocyte autophagy and IL-1ra expression) - Leukocyte senescence	–	Other: - Crystal morphology and surface constituents - Some adaptive immune responses: effector CD41 T cells, IFN-1 - Certain cytokines and protease inhibitors - IL-37 (using MerTK signaling) - Alpha1 anti-trypsin - Some sequelae of PMN activation - Aggregated NET formation - Apoptosis, efferocytosis (including effects of AnnexinA1, Clec12a) - C5a-induced PMN microvesicles (via MerTK signaling)	–

*AMPK* AMP-activated protein kinase, CARD8, Caspase recruitment domain-containing protein 8, *Clec12a* inhibitory C-type lectin receptor 12a, *GM-CSF* granulocyte macrophage-colony stimulating factor, *HDAC* histone deacetylase, *IFN-1* type I interferon, *IL-1ra* IL-1 receptor antagonist, *MerTK* Mer tyrosine kinase receptor for phosphatidylserine, *NET* neutrophil extracellular trap, *PPAR* Peroxisome Proliferator-Activated Receptor γ co-activator 1β (PPARGC1B), PMN neutrophil

## Endogenous and exogenous promoters of gouty inflammation

**Fig. 1:**
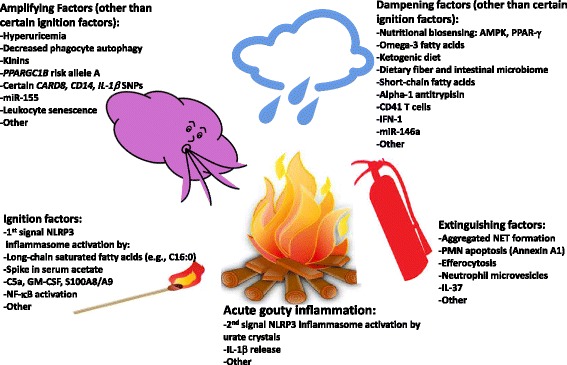
Proposed new model of fundamental factors determining the variability in timing, extent, and duration of acute inflammatory responses to tissue urate crystal deposits. The schematic depicts multiple, recently described mediators of the phenotype of acute gouty inflammation that are discussed in detail in the text, with many cited in Table 1. In this model, acute gouty inflammation is depicted as a fire surrounding a woodpile that is meant to pictorially represent tissue urate crystal deposits. The most recently discovered mediators of ignition of acute gouty inflammation, amplification of the process, and damping and extinguishing of the response are listed in the schematic, cited in further detail in Table 1, and discussed at length in the text. *AMPK* AMP-activated protein kinase, CARD8, Caspase recruitment domain-containing protein 8, *Clec12a* inhibitory C-type lectin receptor 12a, *GM-CSF* granulocyte macrophage-colony stimulating factor, *HDAC* histone deacetylase, *IFN-1* type I interferon, *IL-1ra* IL-1 receptor antagonist, *MerTK* Mer tyrosine kinase receptor for phosphatidylserine, *NET* neutrophil extracellular trap, *PPAR* Peroxisome Proliferator-Activated Receptor γ co-activator 1β (PPARGC1B), PMN neutrophil

Acute gouty arthritis is a prototypical ‘early induced innate immune response’, with periodic, recurrent, short-lived ‘auto-inflammation’, no clear protective immunity to urate crystals, and cytokine-stimulated neutrophil-rich inflammation [8–10]. Neutrophil ingress helps drive robust pain responses [12]. Many different mediators are generated in gouty inflammation, and they can have distinct effects at differing phases of the process to initiate, amplify, dampen, and extinguish acute gout [8–10]. Recent research supports a new hypothetical model of major factors constraining, igniting, and helping to terminate acute gouty arthritis flares, and of multiple additional factors that amplify or otherwise tune the inflammatory phenotype (Fig. 1). Essentially, innate immunity intersects with nutrition, metabolism, and cell fate in phagocytes to shape how the host responds to deposited urate crystals. Individual mediators, and their effects on the gouty inflammation process, are cited in Table 1, and discussed below.

### First signal NLRP3 inflammasome activators

Recent discoveries illuminate the complex alignments of opposing endogenous and exogenous factors at several steps in the gouty inflammation process (Table 1). The canonical NLRP3 inflammasome response to the ‘second signal’, provided by urate crystals, requires ‘first signal’ priming events, including increases in activation of NADPH oxidase and NF-κB, expression of pro-caspase-1, pro-IL-1β, and NLRP3 mRNA, and particle phagocytic capacity [21–23]. Certain first signal NLRP3 inflammasome activators can promote and sustain chronic low-grade inflammation, clinically evident chronic synovitis in gout, and acute inflammatory flares in response to deposited urate crystals. These include C5a [24], GM-CSF [25], and the TLR4 ligand and abundant neutrophil granular protein heterodimer S100A8/A9 (also known as calprotectin) [26]. These mediators are among the factors that further amplify inflammation by promoting phagocyte movement into the inflammatory locus in gout, and activation of phagocytes at those sites (Fig. 1).

Exogenous, dietary-induced, first signal NLRP3 inflammasome activators include long-chain saturated fatty acids such as the TLR2 and TLR4 ligand palmitate [23]; they also appear to include spikes in systemic levels of the short-chain fatty acid acetate [27]. Such acetate spikes can be pronounced (e.g., 20-fold rise) rapidly after alcohol intake. Increased acetate, which signals through the G protein coupled receptor GPR43 [27], could contribute to the association of acute gout flares with high level consumption of all forms of alcohol [28].

### Genetic factors

New findings highlight the genetic impact on the clinical expression of the gouty arthritis phenotype (Table 1), including arthritis flares frequently associated with dietary excesses [29]. The nutritional biosensor peroxisome proliferator-activated receptor (PPAR)-γ is a ligand-dependent nuclear receptor and transcription factor that regulates insulin sensitivity, adipocyte differentiation, and glucose homeostasis. PPAR-γ also exerts a variety of anti-inflammatory effects, in part by transrepression of numerous NF-κB target genes. These effects include suppression of experimental gouty inflammation by some PPAR-γ agonists, mediated in part by suppression of TNF-α [30, 31]. Notably, urate crystals rapidly induce PPAR-γ in monocytes in vitro [31]. However, the natural ligand(s) of PPAR-γ that could limit gouty arthritis remain unclear.

In a study of a Chinese cohort [32], PPAR-γ co-activator 1B (*PPARGC1B*) gene variance permissive for inflammation was increased in those with gout, with the *PPARGC1B* risk A allele rs45520937 approximately doubling the risk of gout. The *PPARGC1B* risk allele A is common (13% in Han Chinese, 4% in Whites) [32]. Transfecting cDNA with this risk allele into cultured human macrophage lineage cells augments inflammatory responsiveness to urate crystals [32].

A small but significant increase in the risk of gout has been linked with functional variants in the inflammasome component caspase activation and recruitment domain (CARD) gene *CARD8*, *IL1B*, and the TLR4 and TLR2 receptor complex co-receptor molecule CD14 [33]. Multiplicative interactions of the gout-associated *IL1B* risk genotype with that of *CARD8* amplify the risk of gout [33]. In contrast, genetic variance in *TLR4* has been linked with gout risk in some populations, but with widely differing results, and the impact is less clear for the heritable variance of TLR4 in gout [34].

### Epigenetic factors

Epigenetic effects are increasingly well understood in the regulation of inflammation, exemplified by the pro-inflammatory effects of some class I histone deacetylases involved in the ability of urate crystals to initiate phagocyte activation [35]. Significantly, phagocyte activation for inflammation is mediated by miR-155. Indeed, peripheral blood mononuclear cells (PBMCs) from gout patients and mouse tissues from experimental acute gout have been shown to have increased miR-155 compared to healthy controls [36]. Urate crystals induce miR-155 in phagocytes and, in turn, elevated miR-155 levels increase the capacity of urate crystals to induce IL-1β and TNF-α; decreased SH2-containing inositol 5’-phosphatase-1 has been shown to be implicated in this mechanism [36].

### Effects of urate levels and crystal morphology

It is well-recognized that ULT can precipitate more acute gout flares. This may be mediated by inflammatory effects through ULT-induced changes in tissue urate equilibrium and remodeling of articular urate crystal deposits, leading to greater accessibility of crystals to inflammatory cells. Essentially, urate crystal deposits formed at different sites within the joint (including synovial fluid, the articular cartilage surface, and within tendons and ligaments, often at insertion sites) have different physical characteristics [37]. Urate crystals at sites of initial nucleation on templates of collagen and proteoglycan-rich connective tissues and urate crystals with a dense protein coating may have a distinct inflammatory potential from crystals at sites of secondary nucleation [37]. It is likely that dynamic changes in urate crystal morphology develop not only with ULT, but also with spikes in the level of hyperuricemia. Moreover, hyperuricemia markedly increases the inflammatory potential of exogenous urate crystals in experimental gout in mice in vivo [38].

A high level of soluble urate, apart from promoting urate crystal deposition, appears to exert a variety of priming effects on inflammation [38–40]. PBMCs of subjects with asymptomatic hyperuricemia were observed to generate greater amounts of multiple inflammatory cytokines than cells from healthy controls when stimulated ex vivo [38, 39]. Moreover, elevated soluble urate suppresses mononuclear phagocyte autophagy and IL-1ra expression and, conversely, activates multiple inflammatory pathways in mononuclear phagocytes, including NF-κB activity and, via the AKT (protein kinase B) pathway and the proline-rich AKT substrate 40 kDa (PRAS 40), activity of the mammalian target of rapamycin [38, 39]. Soluble urate may also activate the NLRP3 inflammasome under hypoxic conditions [41]. However, the impact of hyperuricemia alone on inflammation requires further investigation. In this context, physiologic concentrations of soluble urate have been suggested to have anti-inflammatory as well as chondroprotective effects [42].

### Leukocyte senescence

Replicative senescence not only develops with aging, but also with recurring and chronic inflammation. Senescence promotes tissue inflammation, particularly by differentiation to the senescence-associated secretory cellular phenotype [43]. A recent study suggests linkage of gouty arthritis and cardiovascular disease in gout to leukocyte senescence [44]. Specifically, PBMC senescence has been assessed by quantification of telomere length and telomerase activity in a discovery cohort of Dutch patients with gout (and healthy controls), along with a New Zealand replication cohort [44]. Gout patients have significantly shorter telomeres, with telomere erosion being higher at all ages in patients with the disease and being correlated with gout flare frequency [44]. The shortest telomeres are seen in gout patients with cardiovascular disease [44].

## Endogenous and exogenous suppressors of gouty inflammation

### AMP-activated protein kinase (AMPK)

PPAR-γ is one of the various metabolic regulators that influence gouty inflammation. Specifically, AMPK is a nutritional biosensor that promotes healthy metabolic adaptations to stress, including glucose transport, insulin sensitization, lipid metabolism including β-oxidation of fatty acids, mitochondrial function and biogenesis, balanced cell growth, and autophagy [45]. AMPK is not simply a metabolic ‘super-regulator’ but also suppresses oxidative stress and inflammation [46, 47]. Active AMPK also mediates some of the therapeutic effects of metformin and the anti-inflammatory effects of methotrexate, salicylates, and high-dose aspirin [46]. Activated AMPK markedly suppresses mononuclear phagocyte responses to urate crystals in vitro, including NLRP3 inflammasome activation and IL-1β and chemokine release [47]. AMPK also promotes anti-inflammatory M2 macrophage polarization and autophagy [47]. Genetic AMPK α1 chain deficiency markedly increases mouse inflammatory responses to urate crystals in vivo [47]. Conversely, a pharmacologic AMPK activator suppresses experimental gout-like inflammation in vivo [47].

Caloric deprivation and exercise are among the factors that elevate tissue AMPK activity levels by increasing the cellular AMP:ATP ratio [45, 46]. Conversely, AMPK activity at the tissue level is inhibited by dietary intake of palmitate, fructose, and alcohol excess, as well as by exposure of cells to IL-1β, TNF-α, and urate crystals in vitro [45, 47]. The impact of nutritional biosensing by AMPK on gout is likely substantial in a disease marked by common metabolic co-morbidities and dietary excesses. In this context, obesity, metabolic syndrome, type II diabetes, and hyperglycemia diminish tissue AMPK activity [45]. Moreover, AMPK activity limits progression of several common gout comorbidities, including non-alcoholic steatosis and chronic kidney disease [48].

### Emerging dietary factors

Dietary (e.g., in purine and overall caloric intake) and alcohol consumption restrictions, as well as a reduction in obesity, are dietary measures that have an impact on gouty inflammation [29, 30]. Recently, serum omega-3 fatty acid concentration was suggested to be inversely proportional to the risk of frequent acute gout flares [49]. Omega-3 fatty acids blunt NLRP3-mediated caspase-1 activation and IL-1β release in response to urate crystals and other agonists [50]. Internalization of omega-3 fatty acids by the G protein-coupled receptors 120 (GPR120) and GPR40, and signaling by their downstream scaffold protein β-arrestin-2 (ARRB-2), are centrally implicated in the mechanism [50]. ARBB-2 limits NF-κB activation, and is directly associated with and inhibits NLRP3 [50]. In addition, a ketogenic diet (involving substantial carbohydrate intake restriction), inhibits experimental gouty inflammation via β-hydroxybutyrate and inhibition of NLRP3 inflammasome first-signal priming, as well as IL-1β release from phagocytes [26].

Recent work has suggested beneficial effects of short-chain fatty acids (including acetate and butyrate) in inflammation, including gouty arthritis [35, 51, 52]. Short-chain fatty acids exert a variety of anti-inflammatory effects, mediated in part by limiting leukocyte activation [35]. To date, butyrate is the best studied short-chain fatty acid and is available not simply via intestinal microbiome action on dietary fiber intake, but also from intake of milk fat of grass-eating animals (e.g., cheese, butter, cream). Butyrate inhibits urate crystal-induced expression and release of IL-1β from PBMCs in vitro, transduced by effects including inhibition of certain class I histone deacetylases [35]. Importantly, intestinal microbiome dysbiosis has been reported in two studies of Chinese adults with gout [53, 54]. Fecal material findings reported in gout subjects have included depletion of *Faecalibacterium prausnitzii*, which normally exerts anti-inflammatory effects via butyrate production [53], and differences in several metabolites that modulate inflammation (e.g., increased succinate, which increases IL-1β via modulation of hypoxia inducible factor-1α) [54].

In experimental gout-like inflammation in mice, a high-fiber diet has been reported to induce more rapid resolution, but not onset, of the urate crystal-induced inflammatory response [52]. These results were mimicked by acetate administration, which was effective even after injection of urate crystals into the knee joint, and at the highest level of the inflammatory response [52]. The contributing mechanisms include increased neutrophil apoptosis and inflammation-dampening uptake of apoptotic neutrophils by macrophages (‘efferocytosis’), with associated decreases in NF-κB activity, and increased production of certain anti-inflammatory mediators [52]. Thus, acetate has paradoxical effects on the inflammatory response in experimental gout [26, 27] (Table 1).

### IL-37 and alpha-1 antitrypsin

IL-37, a member of the IL-1 family, is an anti-inflammatory cytokine differentially regulated during the course of tissue inflammatory reactions, largely through expression by epithelial cells and mononuclear leukocytes [55, 56]. IL-37 suppresses multiple innate inflammatory responses in vitro and in vivo, acting partially via inhibition of the NLRP3 inflammasome and activation of suppressor of cytokine signaling 3 [55]. IL-37 actions are mediated partly by the phosphatidylserine receptor Mer receptor tyrosine kinase signaling, and enhanced expression of IL-1R8 [55]. Exogenous human IL-37 markedly suppresses experimental urate crystal-induced inflammation in mice [55]. In a recent study of PBMCs from subjects with acute gout, non-acute gout, non-acute gouty arthritis, and healthy controls, IL-37 was reported as significantly greater in the non-acute gouty arthritis patients compared to acute gout and healthy controls [56], which is in contrast to increases in IL-1β, IL-6, and TNFα in the acute gout group.

Recently, a seasonal, summer drop in the circulating protease inhibitor alpha-1 antitrypsin (and inversely higher stimulated IL-1β production) has been linked with increased summer flares of gout in a large European functional genomics consortium study [57]. Alpha-1 antitrypsin is anti-inflammatory, which acts partly by inducing IL-1ra and inhibiting proteolytic generation of IL-1β, including in response to urate crystals [58]. Moreover, the recombinant human alpha-1 antitrypsin-IgG1 Fc fusion protein markedly inhibits joint inflammation in experimental acute gouty arthritis in mice [58].

### Possible role of adaptive immunity (type I interferon and CD41 T cells)

A fundamental endogenous mechanism limiting the NLRP3-mediated inflammasome pathway in vitro and in vivo is cognate adaptive immunity, which acts via effector and memory CD41 T cells and cell-to-cell contact, and is mimicked by stimulation with selected TNF family ligands (e.g., CD40L) [59]. Moreover, type I interferon suppresses NLPR3 inflammasome activation and IL-1β release [60]. As such, some adaptive immune responses might help maintain clinical inflammatory quiescence of subcutaneous tophi, in which granulomatous structures have been described to have a coronal ring that includes T cells surrounding a central core of massed crystals [61]. The presence of B cells and plasma cells in the tophus corona zone suggests that some form of cognate adaptive immunity could exist in tophi [61].

### NETosis, neutrophil microvesicles, and apoptosis

Recent research has elucidated that neutrophils are required not only to fuel experimental gouty inflammation but also to limit it in vivo [24, 62, 63]. First, at high neutrophil densities achievable in acute gouty arthritis, urate crystals stimulate extracellular trap formation (NETosis) and aggregation [62]. IL-1β is among the stimuli that can induce NETosis. Generally pro-inflammatory, NETosis promotes endothelial damage and thrombosis, in part via release of neutrophil elastase in the extracellular web enriched in chromatin, and by antimicrobial and inflammatory proteins [20, 64]. Complex aggregated NET structures develop in gouty joint fluids and tophi, and aggregated NET formation appears to promote resolution of acute neutrophilic gout-like inflammation in mice. Further, aggregated NET formation may also promote clinically quiescent tophus deposition by trapping and degrading several inflammatory mediators [62]. Conversely, failure of clearance of circulating urate microaggregates in phagocytes has been suggested to promote NETosis, which is toxic to the vasculature [20]. Second, the neutrophil chemotaxin and activator C5a have been reported to induce neutrophil-derived phosphatidylserine-positive microvesicles early in the course of inflammation, including peritonitis induced by urate crystals in mice [24]. Such microvesicles suppress C5a priming of the NLRP3 inflammasome and induction of IL-1β release and neutrophil influx via Mer receptor tyrosine kinase signaling [24]. These effects are shared by synthetic phosphatidylserine-containing liposomes and by neutrophil-derived microvesicles isolated from joint fluids of patients with gouty arthritis [24]. Third, annexin A1 promotes acceleration of neutrophil apoptosis and can combine with acetate, transglutaminase 2, and the inhibitory C-type lectin receptor 12a to promote associated efferocytosis and resolution of the acute phase of experimental gouty inflammation [63, 65, 66].

### Epigenetic effects via miR-146a

Increased miR-146a suppresses multiple urate crystal-induced inflammatory responses in vitro, including IL-1β expression [67]. In a study of patients with gout, miR-146a has been reported to be significantly increased in PBMCs between flares compared to controls with normal serum urate, hyperuricemia, and gout patients during acute flares [67]. Moreover, miR-146a expression is broadly detected in tophi [67]. Urate crystals induce miR-146a in cultured mononuclear phagocytic cells [67]. However, tissue levels of this epigenetic transcriptional suppressor of gouty inflammation decrease early in experimental urate crystal-induced inflammation in mice [67].

## Intersecting effects of therapeutics on gouty inflammation

The mechanisms of action of the primary treatment options for prophylaxis and treatment of acute gout clearly intersect with many of the novel inflammation-mediating pathways cited above. Specifically, indomethacin, ibuprofen, and certain other NSAIDs activate PPAR-γ [68].

Corticosteroids, which inhibit transcription of many inflammatory genes, also regulate annexin A1 [69]. However, corticosteroids also paradoxically induce NLRP3 [70], which may contribute to rebound flares of acute gout after steroid therapy is stopped [71]. Low doses of colchicine suppress not simply neutrophil and endothelial function, but also multiple inflammatory effects in macrophages, transduced in large part by colchicine-induced activation of AMPK [47].

With respect to investigational agents, randomized clinical trials of the IL-1β-specific monoclonal antibody canakinumab have demonstrated superiority compared to a single dose of 40 mg triamcinolone acetonide in reducing pain in acute gout and delaying time to next acute gout flare [71]. Canakinumab is approved in Europe as an option for acute gout therapy. Multiple retrospective case series have reported on the efficacy of anakinra (recombinant IL-1ra) in acute gout in many but not all patients [72]. Failure in acute gout (but not for anti-inflammatory flare prophylaxis) of the IL-1 blocker rilonacept may have been due to binding of rilonacept to IL-1ra [73]. Finally, the partial PPAR-γ agonist arhalofenate, which is a uricosuric, has been reported to be associated with a decreased frequency of acute gout flares in comparison to allopurinol in a Phase II randomized controlled trial comparing ULT strategies and flare prophylaxis [74].

## Future directions of research

The emerging research reviewed here provides new concepts and targets that could identify serum and circulating leukocyte biomarkers for the gout inflammatory state. Circulating IL-8/CXCL8 has potential as a unique cytokine biomarker for gout in the period between flares [75]. In contrast, neither routine acute phase reactants nor serum urate level have a clear role in assessing the inflammatory state in gout, exemplified by increased early flares in ULT and continuing chronic flares in many despite a sustained urate at the desired levels. Several targets reviewed here could be ripe not only for development as biomarkers of the baseline inflammatory state in gout, but also for the clinical testing of new, selective, and safer options for anti-inflammatory prophylaxis and therapy of gout. Of great interest is the emerging generation of orally bioavailable, small molecule NLRP3 inflammasome inhibitors and inhibitors of NLRP3 inflammasome amplification pathways [6, 76]. Direct, potent, and selective AMPK activators and IL-37 also have substantial potential. Other mediators with likely impact, but not adequately investigated to date as gout flare suppressors, include lipoxins and maresins, as well as protectins and resolvins derived from certain omega-3 fatty acids [77].

The potential impact on atherosclerosis in gout and hyperuricemia of circulating urate microaggregates in leukocytes and associated NETosis [20] remains unclear. For example, gout comorbidities and systemic inflammation with expression of pro-atherogenic cytokines IL-1β, IL-8/CXCL8, and IL-6 have the capacity to accelerate atherogenesis. An emerging line of investigation is linking inflammation and heritable urate transporter function in gout [78]. Specifically, the functional Q141K variant of the urate transporter ABCG2, which is strongly associated with hyperuricemia and gout, and compellingly with early onset and tophaceous gout, may inhibit autophagy and modulate systemic inflammation [78]. Interestingly, certain small molecules, including colchicine, invert decreases in ABCG2 141 K cell surface localization and function [78]. Finally, it will be interesting to ascertain whether the high frequency of nocturnal onset of acute gout flares [79] is driven by decreases in endogenous inflammation suppressors beyond altered activity of the hypothalamic-pituitary-adrenal corticosteroid axis.

## Conclusions

Multiple recent discoveries have revealed that urate crystals form at a variety of unexpected sites, but without clear inflammatory sequelae. Work in the last few years has elucidated factors involved in the marked, inherent variability of responses to urate crystals and onset, extent, and duration of flares of acute gouty inflammation. Knowledge of how innate immunity intersects with diet, metabolism, the microbiome, genetics and epigenetics, and phagocyte fate in shaping inflammatory responses to urate crystal deposits in gout provides valuable lessons about inflammation, as well as potential new biomarkers and targets for therapy in gout.
